# Yeast as a tool to decipher the molecular mechanisms underlying the functions of Bcl-2 family

**DOI:** 10.37349/etat.2022.00076

**Published:** 2022-04-02

**Authors:** Stéphen Manon

**Affiliations:** UMR5095, CNRS, Université de Bordeaux, 33077 Bordeaux, France; Regina Elena National Cancer Institute, Italy

**Keywords:** Apoptosis, Bcl-2 family, programmed cell death, yeast, heterologous expression

## Abstract

The budding yeast *Saccharomyces cerevisiae,* a favorite model in biology, does not contain any protein of the Bcl-2 family. From initial experiments with two-hybrid systems to the heterologous expression of human Bcl-2 family members, and the characterization of several forms of yeast programmed cell death, it has however always been a powerful tool to gain information on the mechanisms of apoptosis in general and on Bcl-2 family in particular. This is a short survey of 25 years of experiments that have provided, and at times initiated, insights into the molecular mechanisms underlying the function of Bcl-2 family members.

## Introduction

Apoptosis is the main form of programmed cell death (PCD) in animals. Beyond its crucial functions during development, it plays a central role in the maintenance of tissue homeostasis. As a matter of fact, alterations of the apoptotic process are one of the early characteristics of tumor cells. Furthermore, the efficiency of anti-cancer therapies depends on the apoptotic response of the cells. Hence, defects in the mechanisms of apoptosis are responsible for the failure of these treatments.

Classical anticancer therapies, such as radiotherapy or anti-proliferation chemotherapies, target DNA maintenance and replication. DNA alterations trigger the expression of transcription factor p53, which acts as a tumor suppressor by promoting both cell cycle arrest and apoptosis. A major target of p53 is the Bcl-2 family. The first member of this family, Bcl-2, was identified in 1984 in B-cell lymphomas (hence its name) where its over-expression is the main cause of increased survival [1]. Since then, homologs of Bcl-2 have been identified on the basis of homologies in 4 domains, called BH1 to BH4 [2]. Some of these proteins, like Bcl-2, are anti-apoptotic (Bcl-xL, Mcl-1, A1/Bfl-1, et al.) while others are pro-apoptotic (Bax, Bak, Bok). Other proteins contain only the BH3 domain and are regulators of both anti-apoptotic and pro-apoptotic proteins. Among them, the protein Bid is a close homolog of Bcl-2, while the others have only the BH3 domain in common (e.g., Bim, Bad, Puma). It is noteworthy that, later, proteins having a distantly related BH3 domain have been identified both in mammals and other organisms, where they have functions that are not directly related to apoptosis [3]. However, under certain conditions, these proteins may interact with Bcl-2 family members: for example, the autophagy effector Beclin-1/Atg6 was initially identified as an interactor to Bcl-2 (hence its name) [4, 5].

The main role of Bcl-2 family members is to modulate the permeability of the outer mitochondrial membrane (OMM): pro-apoptotic proteins Bax and Bak are able to form large pores, that promote the release of proteins from the intermembrane space (IMS) towards the cytosol or the nucleus [6, 7]. At least 5 released proteins playing a role in apoptosis have been identified: apoptosis inducing factor (AIF) [8], cytochrome c [9], smac/diablo [10], Omi/HtrA2 [11], and endonuclease G [12]. Released cytochrome c interacts with the protein APAF-1 to form the apoptosome, that initiates the cleavage/activation of procaspase 9 to caspase 9. Once active, caspase 9 cleaves and activates other caspases, namely caspases 3 and 7, that cleaves different substrates. This process has been named the “caspases cascade” [13–15]. Caspases can be inhibited by death regulators, such as the IAPs, that promote caspase degradation by the proteasome. IAPs are themselves inhibited by smac/diablo and Omi/HtrA2 that act by “trapping” them, thus preventing their interaction with caspases. AIF and endonuclease G do not modulate caspases, but act downstream, directly on nuclear DNA degradation. The anti-apoptotic protein of the Bcl-2 family is generally considered to act early in the process by preventing the localization and/or activation of Bax and Bak on the OMM.

This rapid descriptive summary of apoptotic events triggered by anticancer treatments shows that mitochondria are at the heart of the process. It is sometimes written that OMM permeabilization is a “point of no return” of apoptosis. This might be somewhat exaggerated, since caspases activation may still be blocked by IAPs. Nevertheless, the action of the Bcl-2 family on mitochondria is a major step of the apoptotic process, and its deregulation is a major cause of antitumor treatments failure [16–18].

The budding yeast *Saccharomyces cerevisiae* is a favorite model in biology. It can be grown rapidly and easily in every laboratory with minimal equipment. It displays all the basic functions of a eukaryotic cell, with a large set of proteins that are greatly conserved in mammals: it is estimated that 30% of human genes involved in pathologies have a homolog in yeast. Genetic studies are greatly facilitated by its compact genome, with almost no intron (only 4% of yeast genes are spliced after transcription) and the genuine ability of yeast to achieve homologous recombination: yeast can then be “humanized”, by replacing a gene with its human ortholog. It is also easy to maintain non-yeast genes on replicative plasmids that are maintained when yeast divides, or to integrate them at a pre-selected positions in the genome. Last but not least, yeast is very useful for mitochondrial studies: indeed, when mitochondria are deficient, yeast cells are still able to grow owing to alcoholic fermentation, thus providing a way to isolate and study altered mitochondria. For these different reasons, yeast has been used by different investigators to identify new potential functions and targets involved in apoptosis regulation that might be further tested in mammalian cells. In this short survey, we will show several examples of different types of experiments that have been done over the years.

## Yeast as a tool to identify new players in apoptosis

### Identification of new partners of known proteins by the yeast two-hybrid method

The two-hybrid method has been developed in 1989 to test the interaction between any couple of proteins expressed in yeast [19]. It is based on the reconstitution of the transcription factor GAL4 of which the DNA-binding domain and the activation domain are fused to the two proteins of interest. The interaction of the proteins of interest brings the domain together, and the activity of the reconstituted transcription factor can be followed by the capacity of the cells to grow on galactose, or by a colorimetric method with a GAL1-*lacZ* fusion. The method has been improved over the years, with other transcription factors such as LexA [20], and is now a classic element of the molecular biology toolbox.

As early as 1993, a two-hybrid was used to show interactions between Bcl-2 and the protein R-ras p23, which was next confirmed by co-immunoprecipitation [21]. Soon after, the interaction between Bcl-2 family members was evidenced by the two-hybrid approach [22]. The method was then used to investigate which domains and residues of these proteins were involved in the interactions [23, 24]. However, by serendipity, these experiments also established that Bax was able to hamper yeast growth while anti-apoptotic proteins Bcl-2, Bcl-xL, and Mcl-1 prevented this inhibition. Consequently, the investigators quickly moved to the utilization of yeast to identify factors that could modulate the effects of Bcl-2 family members on yeast growth and viability (see below).

Nevertheless, the two-hybrid system remained a method of choice to identify new interactants of known apoptosis regulators. Owing to the specific interactions between BH domains, many BH3-containing proteins were identified through their interaction with Bcl-2 by two-hybrid methods. The most important is the major cell death regulator Bad, identified as a Bcl-2 interactant [25]. The BH3-containing protein BNIP3 was found to interact with Bcl-2 and Bcl-xL and also with the *C.elegans* Bcl-2 homolog Ced-9 [26]. The BH3-containing protein MAP-1, later renamed MOAP-1, was identified as a Bax interactant [27]. More recently, the protein Bcl2L12 was identified through its interaction with both Bcl-2 and Bcl-xL [28]. Other BH3-containing proteins were identified through their interaction with apoptosis regulators that are not Bcl-2 family members, such as NBK/Bik, which was identified as an interactant to the viral protein E1B 19K [29]. Although it is not directly involved in apoptosis, but rather in autophagy, the BH3-containing protein Nix was identified in a two-hybrid screen against a neurotrophin receptor involved in Bax-dependent apoptosis [30].

Other proteins, unrelated to Bcl-2 family members but still able to interact with them, were also identified by two-hybrid, such as the protein Btf [31] and the endophilin-related protein SH3GLB [32]. The two-hybrid system can also be combined with other methods of interaction measurements, such as co-immunoprecipitation, to refine the characterization of hits [33].

Outside from the Bcl-2 family, two-hybrid allowed to identify players of the caspases cascade, such as a new substrate of caspase-7 [34], an inhibitor of APAF-1-driven caspase-9 activation [35], or a regulator of XIAP [36]. Note that, conversely, Bcl-2 family members have also been found as hits in two-hybrid screens with other cancer-related proteins as baits [37].

The general approach used in the studies cited above was to choose an adequate bait (anti- or pro-apoptotic Bcl-2 family member, or any apoptosis effector/regulator of interest) and to screen a cDNA library of possible interactants from healthy or tumor mammalian cells. The main limitations of this approach are the “false positive” responses. Indeed, two-hybrid might be so sensitive that non-specific interactions might be detected. This namely happens in the case of proteins having a hydrophobic α-helix that may interact with any other hydrophobic α-helix. This led to the later-questioned observation that Bax could physically interact with the mitochondrial inner membrane adenine nucleotides transporter (ANT) [38]. To avoid this bias, most investigators choose to work with Bcl-2 family members deprived of their C-terminal hydrophobic α-helix. However, this might have contributed to underestimating the function of this helix in the regulation of Bcl-2 family members, by considering it only as a membrane anchor and a burden for this type of assays.

### Identification of new partners regulating the effects of mammalian proteins in yeast

Since the initial report that the expression of Bax constructs inhibited yeast growth and that this inhibition could be prevented by the co-expression of Bcl-2 or Bcl-xL constructs [22], the investigators found in this unexpected observation a powerful way to identify new regulators of apoptosis. The inhibition of growth was simply measured by drop-tests or replica-plating on a Bax-inducing medium (such as a medium containing galactose when Bax was expressed under the control of the *GAL1/10* promoter) and the restoration of growth was measured on the same medium when Bcl-2 or Bcl-xL were expressed under the same *GAL1/10* promoter or a constitutive promoter, such as *ADH1*. This growth/no-growth phenotype led to some confusion in early papers where the absence of growth was interpreted in terms of cell death [22] while other authors discriminated against the absence of growth and actual cell death [39]. It is now widely accepted that the two phenotypes should be evaluated independently through adequate methods, the more accurate being plating efficiency [40]. Both absorbance or fluorescent molecular probes aiming at differentiating between growing cells, not growing cells, and dead cells are also widely used, but care must be taken that the outcome signal reflects the status of the cells.

The power of studies in yeast can be illustrated through the Bax-induced release of cytochrome c. The demonstration that cytochrome c was released from mitochondria [41] and that cytosolic cytochrome c was required for the apoptotic program [9] was published in 1996. One year later, the demonstration that anti-apoptotic Bcl-2 [42] and Bcl-xL [43] could block cytochrome c release, thus preventing apoptosis, was published. However, the first demonstration that pro-apoptotic Bax directly induced the release of cytochrome c was obtained through the expression of Bax in yeast [44], even before a similar demonstration was done in mammalian cells [45].

**Figure 1.**
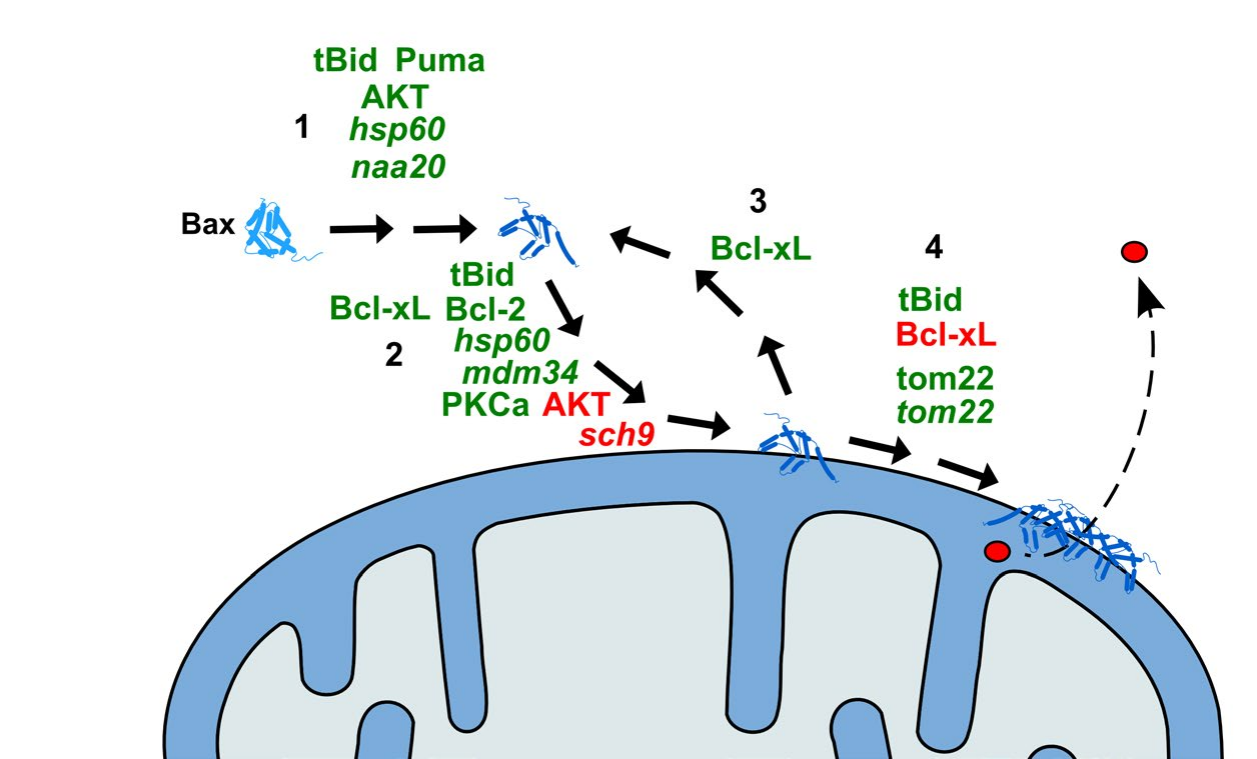
Schematic representation of the action of modulators of Bax effect on yeast mitochondria. 1: Activation of Bax (conformational change); 2: translocation of Bax to the MOM; 3: retrotranslocation of Bax from the MOM; 4: oligomerization of Bax and formation of the pore. Only the proteins of which the direct effect on Bax has been demonstrated are represented. Activators of the relevant step are in green; inhibitors are in red. Names in italic are yeast proteins

Although it does not accurately reflect cell death, the clarity and the practicality of the no-growth phenotype induced by Bax expression in yeast opened the way to the identification of suppressors. The first obvious suppressors were the anti-apoptotic members of the Bcl-2 family [22, 46–49]. But this has been extended to other mammalian proteins, through the identification of suppressors of Bax (or Bak)—induced growth impairment [50–58] (Table 1 and Figure 1).

**Table 1.** An overview of proteins that have been shown to modulate Bax-induced cell death in yeast

**Function**	**Activation: overexpression stimulates Bax effects and/or deletion inhibits Bax effects**		**Inhibition: overexpression inhibits Bax effects and/or deletion stimulates Bax effects**		
	**Mammalian proteins**	**Yeast proteins**	**Mammalian proteins**	**Yeast proteins**	**Plant and viral proteins**
Bcl-2 family and related	tBid [171, 172] Puma [173] Noxa, Bik [174]		Bcl-2, Bcl-xL [22, 39, 44, 46, 175] Bfl-1/A1 [176]	see below for Ybh3	M11 [177] DPV022 [178]
Bax-inhibitor			BI-1 [50]	BI-1/Bxi1/Ybh3 [59] (not tested with Bax in [89, 90])	BI-1 [59, 61, 179, 180]
Traffic	Tom22 [135, 137]	Tom22 [135, 139] TOM complex [136] Mdm34 [148]	Vps34 [54] Vamp3 [181]	Sec22 [182]	AtVAMP [63]
Autophagy and protein quality control		Uth1 [73, 183] Yme1 [184] Naa20 [185]		Atg5, Atg7 [73]	Sentrin [186]
Protein aggregates			PrP [187,188] α-synuclein [189]		
Protein- kinases	AKT [128] PKCα [190]		AKT [128]	Sch9 [191]	
Stress responses		Hsp60 [192] Hog1 [193]	Trx1 [194] Fth1 [195]	Tsa1 [194] Rgi1 [195]	tQM [196] LePHGPx [64] AtEBP [62, 65] BI-GST [60, 197] sAPX [198]
Energetic metabolism		Qcr7, Cyc3, Cox4, Cox7, Atp4, Pet9 [161, 199] Data not confirmed in [155]	COX6A1 [200]	OYE2 [69, 201] Por1 [199]	GAPDH [202]
Lipid metabolism			SMS1 [203]	Hfd1 [184] Fah1 [204]	fah1, fah2 [204] BcLCB(2) [205]
Others			hRPL9 [206] Septin7 [70] Tsc22 [55]	Cdc10 [70]	Omp2b [67]

Conversely, it is theoretically possible to identify suppressors of the anti-apoptotic function of Bcl-2 or Bcl-xL. However, this is more difficult since it implies the use of a negative screening (i.e., finding clones that lose their ability to grow when Bcl-2 or Bcl-xL is co-expressed with Bax) by replica plating. To our knowledge, no successful large-scale assay has been published to date.

Because a large number of functions and regulations are conserved between yeast and higher eukaryotes, the identification of Bax suppressors (or Bcl-2/Bcl-xL suppressors) is not limited to mammalian genes. The screening of libraries from invertebrates [59] and plants [59–67] allowed to identify proteins relevant to cell death that might have not been previously identified in mammals, with the noticeable exception of the protein Bax inhibitor-1 that has been found in every organism tested [68]. cDNA from the yeast itself has been used and several genes involved in yeast resistance to Bax have been identified this way [69, 70].

It is necessary to point out, however, that these experiments may display a major bias, related to the way Bax prevents yeast growth or even induces yeast cell death. As we will see in the next chapter, moderate oxidative stress induces a form of yeast PCD that exhibits several similarities to mammalian apoptosis. It has been shown that Bax expression in yeast actually induces moderate oxidative stress [71, 72]. Furthermore, Bax-induced yeast growth inhibition might not be related to PCD but rather to autophagy [73]. This might indicate that the protective effect of over-expressed mammalian, plant, or yeast genes over Bax effects in yeast, might not be fully related to Bax itself but rather to downstream events specific to yeast. It is therefore crucial to complete the identification of Bax suppressors by experiments in the original organism before being conclusive. Yeast is such a powerful tool that we, investigators, should not forget that it is only a tool.

### Identification of suppressors of yeast PCD

The first evidence that yeast displays a form of PCD having common features with apoptosis was obtained in a mutant of the cell cycle protein cdc28 [74] and, rapidly after, it was observed that Bax expression in yeast induced similar features [75]. It was then observed that (moderate) oxidative stress was a major regulator of yeast PCD [76]. It followed that any alteration producing moderate oxidative stress (including Bax expression, as we cited above) could induce yeast PCD having some similarities to mammalian apoptosis. This included acetic acid stress [77], high salt concentration [78], α-mating pheromone [79], amiodarone [80], aging [81], defects in *N*-glycosylation [82], among others (see [83] for a recent exhaustive survey).

The large collection of available yeast deletion mutants has allowed identifying proteins involved in this form of cell death. The main interest is that several of these proteins have homologs in mammals. For example, the deletion of the gene *SRO7*/*SOP1* induced a loss of viability, with several markers of apoptosis, when the cells were grown in the presence of high NaCl concentration and, interestingly, this protein is the homolog of the *Drosophila l(2)gl* tumor suppressor [84]. HIR1, a co-repressor of histone transcription, acts as a suppressor of yeast cell death induced by various stress, such as mRNA stabilization and acetic acid treatment [85]. The human homolog of HIR1, HIRA, had been previously associated with different apoptosis alterations during development [86]. Studies in the fission yeast *Schizosaccharomyces pombe* have shown that calnexin is involved in apoptotic cell death induced by endoplasmic reticulum (ER) stress [87]. A genetic screen identified HMG1/2, the homolog of the human protein HMGB1, as a suppressor of both yeast and human calnexin-induced apoptosis, showing that this death pathway is largely conserved between fission yeast and human cells. Like for Bax-induced cell death, a large number of proteins having direct or indirect effects on oxidative stress have been identified as suppressors, such as PGK1 [88].

A particular interest has been focused on the protein called Ybh3, identified as a BH3-containing protein (hence its name, for yeast BH3) [89]. Beyond the discussion on whether or not the BH3 domain of this protein is a genuine one [3], it was particularly intriguing that this protein, that favors cell death, has been simultaneously identified as an anti-apoptotic protein [90], and is actually a homolog of the widely characterized Bax inhibitor identified in plants and animals [91, 92]. The identification as pro-apoptotic protein was done in cells submitted to moderate oxidative stress, while the identification as anti-apoptotic proteins was done on cells submitted to ER stress. It is noteworthy that several Bcl-2 family members may have opposite functions, depending on different modifications. A shorter splicing variant of Bcl-xL, named Bcl-xS, was identified at the same time and was shown to be pro-apoptotic [93]. Caspase-3-mediated cleavage of Bcl-2 was shown to generate a ΔN34 variant that permeabilized mitochondria to cytochrome c [94]. Conversely, the phosphorylation of Bax on Ser184 not only prevented Bax mitochondrial localization (see the discussion below) but could, under certain conditions, convert Bax into an anti-apoptotic protein through its binding to BH3-only proteins [95]. There is no indication, to date, that such processes occur for yeast Ybh3, but this might be a trail to explore its apparent dual function.

Yeast PCD also involves proteins that have been identified as homologs of known mammalian apoptosis regulators. The most obvious is cytochrome c, a universal and highly conserved mobile electron transporter [96]. In non-apoptotic eukaryotic cells, it is localized in the mitochondrial IMS but remains in close proximity of the inner membrane, more specifically of respiratory complexes III and IV. When released on the cytosol during apoptosis, cytochrome c interacts with APAF-1 to form the apoptosome, in a process that depends on the conserved K72 residue [97]. No yeast homolog of APAF-1 has been identified to date and the role of cytochrome c in yeast cell death, if any, remains unclear. It can be speculated that cytosolic cytochrome c contributes to the modulation of the intracellular redox status [98] but this has never been clearly supported.

Yeast does not contain caspase but expresses a metacaspase, called Yca1. Its deletion delays or decreases physiological cell death induced by many stimuli [99], but not by Bax [73]. The actual role of this enzyme in yeast cell death has been and still is a matter of debate [100]. Other homologs of apoptogenic factors, such as yeast AIF [101], endonuclease G [102], and Nma111/Omi/HtRA2 [103] have been identified as regulators of several physiological yeast death pathways. Yeast AIF is not involved in Bax-induced death [73] and the two others have not been investigated to date.

Beyond the study of apoptotic mechanisms *senso strictu*, yeast can be a powerful tool to study the connections between PCD and the regulation of growth and survival processes such as cell cycle [104], autophagy [104, 105], RNA stability [106], and, more generally, metabolism [107–109]. These processes are generally better conserved than PCD, between yeast and mammals. The information gained from studies in yeast, either from “physiological” yeast cell death or from “ectopic” cell death induced by mammalian proteins can therefore bring useful information on cross-talks between PCD and these processes in mammalian cells.

However, extrapolations from yeast to mammals should be done with great care. For example, two major autophagy regulators in mammalian cells have a BH3 domain: Beclin-1, which is involved in the initiation of the formation of autophagosomes [110], and Bcl2L13, a *bona fide* Bcl-2 family member, that is the mitochondrial receptor of mitochondria-targeted autophagy [111]. Besides their role in autophagy, these two proteins are able to regulate (and be regulated by) Bcl-2 family members [112, 113]. Their yeast homologs, Atg6 and Atg32, do not have a BH3 domain. Two speculations can be made: (i) autophagy/apoptosis cross-talks that exist in mammals do not exist in yeast or (ii) autophagy/apoptosis cross-talks in yeast do not require any BH3 domain (because there are no genuine Bcl-2 family members in yeast).

These examples underline that yeast PCD should be considered as a biological process that deserves to be studied somewhat independently from mammalian apoptosis. Both processes clearly share some similarities, some signaling pathways are undoubtedly homologous to the point that human proteins can compensate for the absence of yeast proteins, but the two processes of yeast PCD and mammalian cell death have evolved in parallel but not identically [40]. The obvious rationale behind this distinct evolution is probably the fact that yeast is a unicellular organism. It might exhibit some cooperative features [114]. An outstanding study further demonstrated the role of yeast PCD in the organization of yeast colonies [115, 116]. But it remains that yeast PCD does not have the same *raison d'être* as mammalian PCD and it must be kept in mind that they are not the copy of each other.

## Yeast as a tool for structure/function studies of Bcl-2 family members

The initial observation that Bax constructs were able to inhibit yeast growth and that this inhibition was prevented by Bcl-2 and Bcl-xL [39, 117] opened the way to the utilization of yeast as a tool to investigate further the mechanistic events underlying the action of the Bcl-2 family members on mitochondria. Indeed, yeast provided a “living test tube” to investigate the function of a limited number of proteins, thus simplifying the interaction network. As we already noted, Bax expression in the yeast provided the first demonstration that the protein was directly responsible for cytochrome c release [44]. Furthermore, Bax expression in yeast complemented studies on mammalian mitochondria to show that Bax alone was able to form a large pore in the OMM, having a size compatible with the release of cytochrome c [118].

Hence, our group and several others have identified domains and residues involved in the intricate process of Bax mitochondrial relocation and activation. The first question was about the actual role of the C-terminal hydrophobic α-helix of Bax as a membrane anchor. This was not a trivial question since, contrary to Bcl-xL, purified Bax was not able to bind to isolated mammalian mitochondria. Furthermore, the replacement of the C-terminus of Bax by the C-terminus of Bcl-xL generated a chimera Bax-CxL that was able to bind to isolated mitochondria, while the reverse construction did not [119]. When expressed in yeast, truncated Bax was able to permeabilize the OMM to cytochrome c but lost its sensitivity to the inhibition by Bcl-xL [46]. The chimera Bax-CxL was still localized to mitochondria both in mammalian and yeast cells, but lost its pro-apoptotic properties, showing that the mitochondrial localization of Bax is not sufficient to promote the permeabilization [47, 120]. Substitutions of residues in the Bax C-terminus confirmed that its hydrophobic nature was not crucial for Bax mitochondrial localization, but that its movement out from the hydrophobic groove formed by the BH domains was a crucial step in its activation, that could be mimicked by the single substitution P168A in yeast [121], in mammalian cells [122] and in liposomes [123].

Part of the regulation of this movement relies on the possible phosphorylation of residue S184. It was demonstrated that the deletion of this residue generated a membrane anchor much more “efficient” than the genuine hydrophobic α-helix [124]. The identification of S184 as a target of several protein kinases, including the survival kinase AKT [125] raised the hypothesis that the phosphorylation of this residue could control the movement of the C-terminal helix. The substitution of S184 by non-phosphorylatable (A, V) or phosphomimetic (D, E) residues was in accordance with the hypothesis that S184 phosphorylation limited Bax mitochondrial localization, both in mammalian cells [125] and yeast cells [126]. However, the actual effects on Bax function seem to remain somewhat contradictory [95, 127–129], as a possible consequence of a differential effect of anti-apoptotic proteins on non-phosphorylated or phosphorylated Bax [127, 130].

**Figure 2.**
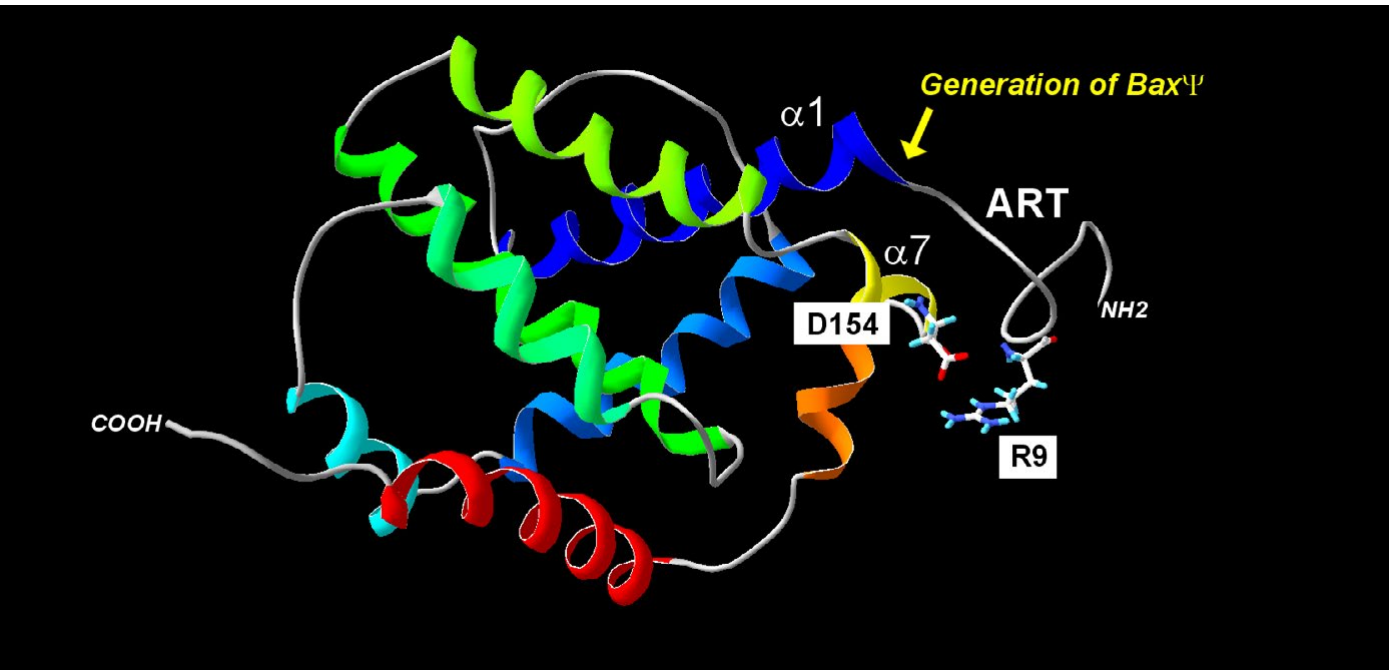
Regulation of Bax mitochondria localization by the ART. The absence of the ART (20 N-terminal residues) generates the BaxΨ variant, which is spontaneously mitochondrial and active. Within the ART, the R9 residue may interact with the residue D154 of helix α7. Point mutations in the helix α1 decrease the mitochondrial localization of both wild-type Bax (BaxWT) and BaxΨ. Together, these data suggest that a movement of ART away from the core of the protein may contribute to the exposure of helix α1 to mitochondrial partners

Another interesting issue that was addressed in yeast, was the role of the N-terminus of Bax (Figure 2; Table 2). Among natural Bax variants found in tumors, an N-terminally truncated mutant called BaxΨ was found in low-grade glioblastoma [131], and this variant was found to be more active than full-length Bax in mammalian cells, in yeast, and in reconstituted systems, leading to the conclusion that the N-terminal end of Bax was a negative regulator of its activity [132, 133]. It was next observed that the domain that follows immediately, corresponding to helix α1 contained residues that were crucial for Bax interaction with mitochondria, suggesting the existence of a mitochondrial Bax receptor [134] that was further identified as Tom22 both in mammalian and yeast cells [135–137]. It is noteworthy that this regulation was lost on isolated mitochondria [138], which might be related to the fact that Tom22 does not act as a *bona fide* mitochondrial receptor to Bax, but rather as a regulator for its insertion under a regulatable conformation [137, 139].

**Table 2.** Mutational analysis of the N-terminus of Bax in yeast and mammalian models [121, 126, 133, 134]

**Sequence**	**Protein**	**Binding to mitochondria**		**Cytochrome c release**	
		**Yeast**	**Mammals**	**Yeast**	**Mammals**
(1) Role of ART and Proline residues at positions 8 and 13					
-P_8_RGGGP_13_-	full length Bax	+/−	+/−	+/−	+/−
	BaxΨ/ΔART	+++	+++	+++	+++
-**G**_8_RGGG**G**_13_-	ART mutant	+++	n.d.	+++	n.d.
-**V**_8_RGGG**V**_13_-	ART mutant	n.d.	+++	n.d.	+++
-**V**_8_RGGGP_13_-	ART mutant	n.d.	+/−	n.d.	+/−
-P_8_RGGG**V**_13_-	ART mutant	n.d.	+++	n.d.	+++
(2) Role of helix α1					
-A_24_LLL_27_-	BaxΨ/ΔART	+++	+++	+++	+++
	BaxΨ/ΔART ΔHα1	n.d.	−	n.d.	−
-**R**_24_LLL_27_-	BaxΨ/ΔART Hα1 mutant	+	−	+	−
-A_24_L**GV**_27_-	BaxΨ/ΔART Hα1 mutant	+++	−	+++	−
(3) Interaction between ART and BH2 (helix α7/8)					
-P_8_RGGGP_13_- -I_152_QDQG_156_-	full length human Bax	+/−	+/−	+/−	+/−
-P_8_**E**GGGP_13_- -I_152_QDQG_156_-	ART mutant	+++	n.d.	+++	n.d.
-P_8_RGGGP_13_- -I_152_Q**K**QG_156_-	BH2 mutant	+++	n.d.	+++	n.d.
-P_8_**E**GGGP_13_- -I_152_Q**K**QG_156_-	ART mutant BH2 mutant	−	n.d.	−	n.d.

(1) The substitution of prolines 8 and 13, like the complete deletion of the 20 N-terminal residues, increase Bax binding and activity both in yeast and mammals; (2) substitutions in helix α1 decrease the binding of BaxΨ in mammals. The decrease is partly visible in yeast; (3) individual point mutations inverting charges in ART and in BH2 induce stimulation of Bax in yeast, but a double charge change revert to the wild-type behavior of Bax, suggesting the existence of an interaction between charged residues in these two domains, stabilizing the inactive conformation. Bold residues indicate mutations. −: inactive; n.d.: not determined; +/−: poorly active; +: active; +++: strongly active

The ART domain contains a positively charged residue, R9, which, given the high mobility of ART can be in relatively close proximity to a negatively charged residue, D154, in the helix α7 (~7Å). The introduction of two negative charges (E9/D154) or two positive charges (R9/K154) generates mutants that have a greater mitochondrial localization (and a greater activity) than BaxWT (R9/D154) or the reverse mutant (E9/K154), suggesting that the interaction between these two residues regulated negatively Bax mitochondrial localization and activity [121].

Strikingly, a recent study showed that Bak, the other pro-apoptotic member of the Bcl-2 family, also supports a negative regulation of its insertion/activation process by a domain located on the N-terminal moiety of the protein [140]. Although the domain and mode of regulation are different, this negative regulation seems to be a conserved feature between the two proteins.

The role of mitochondrial receptors in the localization of Bcl-2 family members, and the utilization of yeast to investigate this role, are not limited to Bax. Yeast mitochondria deficient for Tom20 are less able to bind Bcl-2 [141]. The same study pointed at the critical role of positive charges flanking the C-terminal α-helix of Bcl-2 in its mitochondria addressing, a property that was later confirmed in mammalian mitochondria [142]. More recently, the role of Tom20 was specified, both in mammalian and yeast cells, showing that Tom20 facilitated the ER to mitochondria transfer of Bcl-2 after apoptosis has been initiated and Bax has been activated [143]. The role of Tom70 as a receptor for the BH3-only protein Bim has also been suggested through experiments in yeast, even though the actual role of this interaction remains unclear [144].

Yeast is also a powerful tool to investigate the role of contacts between ER and mitochondria, corresponding to domains called mitochondria-associated membrane (MAM). Indeed, contrary to mammalian cells in which the occurrence of MAM depends on different complexes [145, 146], yeast MAM depends on the well-characterized ER-mitochondrial encounter structure (ERMES) [147]. The deletion of one component of ERMES, the protein mdm34, has been shown to limit the mitochondrial localization of Bax [148] and, as a consequence, of Bcl-2, when both proteins are co-expressed [144].

A most remarkable characteristic of Bax mitochondrial localization is its reversibility, which has been identified by demonstrating that Bcl-xL could retrotranslocate Bax from mitochondria to the cytosol [149]. It was next demonstrated that the C-terminal end of Bcl-xL was required for retrotranslocation [150]. The retrotranslocation of Bax by Bcl-xL was also observed in yeast, where it was further shown that Bcl-xL also stimulated translocation, by a process that did not depend on an intact C-terminal end of Bcl-xL, thus explaining why truncated Bcl-xL greatly increased Bax mitochondrial content [151, 152].

A large number of mitochondrial proteins are conserved between yeast and mammals, and yeast has extensively been used as a simplified model to confirm or rebut suspected mitochondrial regulations of Bcl-2 family members addressing and activating. Voltage-dependent anion-selective channel (VDAC), the channel responsible for the permeability of the OMM to metabolites, has been proposed to be involved in Bax- induced cytochrome c release [153]. However, yeast cells depleted for VDAC isoforms did not respond to Bax differently from wild-type yeast cells [48, 154, 155], and this was later confirmed in mammalian cells [156]. Here again, experiments in simple yeast cells have been more reliable than experiments in more intricate mammalian cells.

Another example of conserved proteins between yeast and mammals is given by chaperones, such as the Hsp70 family that is universally present in prokaryotes and eukaryotes [157, 158]. The BH3-only protein Bim has been shown to interact with mammalian Hsp70 through its BH3 domain [159]. The heterologous expression of Bim in yeast stimulated cell growth and increased the protection against heat shock [160], thus providing a simple model to study the role of Bim as a co-chaperone.

The yeast model is not without inconvenience, however. The ability of yeast to grow without functional mitochondria can generate confusion. For example, it has been observed that a deletion of subunit 4 of mitochondrial FoF1-ATP synthase prevented the effect of Bax on yeast growth, leading to the conclusion that this complex was somehow involved in the Bax effect [161]. However, yeast mutants in ATP synthase are prone to generate cells losing a large portion (when not all) of their mitochondrial DNA, thus lacking all of the respiratory complexes. Yeast cells lacking mitochondrial DNA had been previously shown to survive Bax expression (although their growth rate was affected) likely because of different sensitivity to oxidative stress [38] and this could explain the resistance of the *ΔAtp4* mutant.

Besides mammalian proteins, yeast has also been used as a complementary tool to further characterize the function of newly identified Bcl-2 family members from other organisms such as zebrafish [162] or Trichoplax [163].

## Could yeast be useful for apoptosis-targeting drug screening?

Due to the clarity of the growth/no growth phenotypes linked to the expression of Bcl-2 family members in yeast, it was obviously tempting to use yeast as a rapid “pre-screen” to identify molecules of interest in targeting apoptosis, especially that known molecules had a significant effect on yeast [164]. Since it is easier to identify a drug that suppresses the “no growth” phenotype than the opposite, such screening is expected to be more efficient to identify anti-apoptotic molecules, of potential interest in treating degenerative diseases, than to identify pro-apoptotic molecules, of potential interest in treating proliferative diseases [165].

Several screenings have been successful in identifying caspases activators [166] or p53/mdm2 pathway activators [167, 168]. However, to our knowledge, no successful screening has been published for the Bcl-2 family. The reason for this failure might rely on the fact that the Bax-induced phenotype of growth inhibition/death is complex, involving yeast PCD [75], oxidative stress response [71, 72], and autophagy [73].

This is, however, not without a solution. Owing to genetic manipulation and the plasticity of yeast, it might be possible to limit the “side responses” of yeast by working in an autophagy-deficient or stress response-deficient genetic context. Also, yeast can be used to further characterize the effects of an already known molecule in a simpler model, which allows for confirmation of the relevance of a target. As an example, the BH3-mimetic ABT-737, a well-established inhibitor of both Bcl-2 and Bcl-xL binding to Bax, had been suggested to promote a further activation of Bax because the binding of the anti-apoptotic proteins caused a conformational change of the pro-apoptotic protein, thus promoting its full activation once the binding is challenged by the inhibitor [169]. This “pre-activation” process of Bax by Bcl-xL causing a greater efficiency of ABT-737 was next confirmed in yeast, showing that this process is dependent only on the two proteins, without the involvement of a third partner [151, 152].

## Conclusions

With the outstanding development of genetic tools in mammalian cells, one might think that yeast has lost a large part of its attractivity to study processes such as apoptosis. However, beyond the basic manipulation of genes, which is greatly facilitated by its compact genome and the nearly infinite collection of mutants available in the world, yeast keeps many interests: its ability to grow even though mitochondria are altered, the possibility to study a limited number of proteins and its outstanding capacity to overcome adverse conditions by activating or inhibiting enzymes or whole pathways far away from the initial alteration, thus providing unsuspected possible regulations. Still a vivid tool for geneticists, yeast is sometimes snubbed by some cell biologists and biochemists, who label yeast studies as “old science”. Considering the number of Nobel Prizes awarded to yeast investigators [170], they are likely wrong.

## Abbreviations

AIF: apoptosis inducing factor
ER: endoplasmic reticulum
MAM: mitochondria-associated membrane
OMM: outer mitochondrial membrane
PCD: programmed cell death

## Declarations

### Copyright

© The Author(s) 2022.

## References

[1] Tsujimoto Y Cossman J Jaffe E Croce CM. Involvement of the *bcl-2* gene in human follicular lymphoma. Science. 1985;228:1440–3. 10.1126/science.3874430 3874430

[2] Gross A McDonnell JM Korsmeyer SJ. BCL-2 family members and the mitochondria in apoptosis. Genes Dev. 1999;13:1899–911. 10.1101/gad.13.15.1899 10444588

[3] Aouacheria A Rech de Laval V Combet C Hardwick JM. Evolution of Bcl-2 homology motifs: homology *versus* homoplasy. Trends Cell Biol. 2013;23:103–11. 10.1016/j.tcb.2012.10.010 23199982 PMC3582728

[4] Liang XH Kleeman LK Jiang HH Gordon G Goldman JE Berry G Protection against fatal Sindbis virus encephalitis by beclin, a novel Bcl-2-interacting protein. J Virol. 1998;72:8586–96. 10.1128/JVI.72.11.8586-8596.1998 9765397 PMC110269

[5] Pattingre S Tassa A Qu X Garuti R Liang XH Mizushima N Bcl-2 antiapoptotic proteins inhibit Beclin 1-dependent autophagy. Cell. 2005;122:927–39. 10.1016/j.cell.2005.07.002 16179260

[6] Renault TT Chipuk JE. Death upon a kiss: mitochondrial outer membrane composition and organelle communication govern sensitivity to BAK/BAX-dependent apoptosis. Chem Biol. 2014;21:114–23. 10.1016/j.chembiol.2013.10.009 24269152 PMC3947007

[7] Dadsena S King LE García-Sáez AJ. Apoptosis regulation at the mitochondria membrane level. Biochim Biophys Acta Biomembr. 2021;1863:183716. 10.1016/j.bbamem.2021.183716 34343535

[8] Susin SA Zamzami N Castedo M Hirsch T Marchetti P Macho A Bcl-2 inhibits the mitochondrial release of an apoptogenic protease. J Exp Med. 1996;184:1331–41. 10.1084/jem.184.4.1331 8879205 PMC2192812

[9] Liu X Kim CN Yang J Jemmerson R Wang X. Induction of apoptotic program in cell-free extracts: requirement for dATP and cytochrome c. Cell. 1996;86:147–57. 10.1016/S0092-8674(00)80085-9 8689682

[10] Du C Fang M Li Y Li L Wang X. Smac, a mitochondrial protein that promotes cytochrome c-dependent caspase activation by eliminating IAP inhibition. Cell. 2000;102:33–42. 10.1016/S0092-8674(00)00008-8 10929711

[11] Suzuki Y Imai Y Nakayama H Takahashi K Takio K Takahashi R. A serine protease, HtrA2, is released from the mitochondria and interacts with XIAP, inducing cell death. Mol Cell. 2001;8:613–21. 10.1016/S1097-2765(01)00341-0 11583623

[12] Li LY Luo X Wang X. Endonuclease G is an apoptotic DNase when released from mitochondria. Nature. 2001;412:95–9. 10.1038/35083620 11452314

[13] Kumar S Harvey NL. Role of multiple cellular proteases in the execution of programmed cell death. FEBS Lett. 1995;375:169–73. 10.1016/0014-5793(95)01186-I 7498492

[14] Cohen GM. Caspases: the executioners of apoptosis. Biochem J. 1997;326(Pt 1):1–16. 10.1042/bj3260001 9337844 PMC1218630

[15] Adams JM Cory S. Apoptosomes: engines for caspase activation. Curr Opin Cell Biol. 2002;14:715–20. 10.1016/S0955-0674(02)00381-2 12473344

[16] Reed JC. Dysregulation of apoptosis in cancer. J Clin Oncol. 1999;17:2941–53. 10.1200/JCO.1999.17.9.2941 10561374

[17] Fulda S Debatin KM. Exploiting death receptor signaling pathways for tumor therapy. Biochim Biophys Acta. 2004;1705:27–41. 10.1016/j.bbcan.2004.09.003 15585171

[18] Gatti L Zunino F. Overview of tumor cell chemoresistance mechanisms. Methods Mol Med. 2005;111:127–48. 10.1385/1-59259-889-7:127 15911977

[19] Fields S Song O. A novel genetic system to detect protein-protein interactions. Nature. 1989;340:245–6. 10.1038/340245a0 2547163

[20] Wilson TE Padgett KA Johnston M Milbrandt J. A genetic method for defining DNA-binding domains: application to the nuclear receptor NGFI-B. Proc Natl Acad Sci U S A. 1993;90:9186–90. 10.1073/pnas.90.19.9186 8415675 PMC47527

[21] Fernandez-Sarabia MJ Bischoff JR. Bcl-2 associates with the ras-related protein R-ras p23. Nature. 1993;366:274–5. 10.1038/366274a0 8232588

[22] Sato T Hanada M Bodrug S Irie S Iwama N Boise LH Interactions among members of the Bcl-2 protein family analyzed with a yeast two-hybrid system. Proc Natl Acad Sci U S A. 1994;91:9238–42. Erratum in: Proc Natl Acad Sci U S A. 1995;92:2016. 10.1073/pnas.91.20.9238 7937747 PMC44787

[23] Sedlak TW Oltvai ZN Yang E Wang K Boise LH Thompson CB Multiple Bcl-2 family members demonstrate selective dimerizations with Bax. Proc Natl Acad Sci U S A. 1995;92:7834–8. 10.1073/pnas.92.17.7834 7644501 PMC41240

[24] Ottilie S Diaz JL Chang J Wilson G Tuffo KM Weeks S Structural and functional complementation of an inactive Bcl-2 mutant by Bax truncation. J Biol Chem. 1997;272:16955–61. 10.1074/jbc.272.27.16955 9202007

[25] Yang E Zha J Jockel J Boise LH Thompson CB Korsmeyer SJ. Bad, a heterodimeric partner for Bcl-XL and Bcl-2, displaces Bax and promotes cell death. Cell. 1995;80:285–91. 10.1016/0092-8674(95)90411-5 7834748

[26] Ray R Chen G Vande Velde C Cizeau J Park JH Reed JC BNIP3 heterodimerizes with Bcl-2/Bcl-X(L) and induces cell death independent of a Bcl-2 homology 3 (BH3) domain at both mitochondrial and nonmitochondrial sites. J Biol Chem. 2000;275:1439–48. 10.1074/jbc.275.2.1439 10625696

[27] Tan KO Tan KM Chan SL Yee KS Bevort M Ang KC MAP-1, a novel proapoptotic protein containing a BH3-like motif that associates with Bax through its Bcl-2 homology domains. J Biol Chem. 2001;276:2802–7. 10.1074/jbc.M008955200 11060313

[28] Yang MC Loh JK Li YY Huang WS Chou CH Cheng JT Bcl2L12 with a BH3-like domain in regulating apoptosis and TMZ-induced autophagy: a prospective combination of ABT-737 and TMZ for treating glioma. Int J Oncol. 2015;46:1304–16. 10.3892/ijo.2015.2838 25586056

[29] Han J Sabbatini P White E. Induction of apoptosis by human Nbk/Bik, a BH3-containing protein that interacts with E1B 19K. Mol Cell Biol. 1996;16:5857–64. 10.1128/MCB.16.10.5857 8816500 PMC231587

[30] Shen J Chen X Li H Wang Y Huo K Ke K. p75 neurotrophin receptor and its novel interaction partner, NIX, are involved in neuronal apoptosis after intracerebral hemorrhage. Cell Tissue Res. 2017;368:13–27. 10.1007/s00441-016-2510-y 27726026

[31] Kasof GM Goyal L White E. Btf, a novel death-promoting transcriptional repressor that interacts with Bcl-2-related proteins. Mol Cell Biol. 1999;19:4390–404. 10.1128/MCB.19.6.4390 10330179 PMC104398

[32] Pierrat B Simonen M Cueto M Mestan J Ferrigno P Heim J. SH3GLB, a new endophilin-related protein family featuring an SH3 domain. Genomics. 2001;71:222–34. 10.1006/geno.2000.6378 11161816

[33] Wong C Naumovski L. Method to screen for relevant yeast two-hybrid-derived clones by coimmunoprecipitation and colocalization of epitope-tagged fragments--application to Bcl-xL. Anal Biochem. 1997;252:33–9. 10.1006/abio.1997.2284 9324938

[34] Araya R Takahashi R Nomura Y. Yeast two-hybrid screening using constitutive-active caspase-7 as bait in the identification of PA28gamma as an effector caspase substrate. Cell Death Differ. 2002;9:322–8. 10.1038/sj.cdd.4400949 11859414

[35] Chau BN Cheng EH Kerr DA Hardwick JM. Aven, a novel inhibitor of caspase activation, binds Bcl-xL and Apaf-1. Mol Cell. 2000;6:31–40. 10.1016/S1097-2765(05)00021-3 10949025

[36] Zheng ZL Tan LZ Yu YP Michalopoulos G Luo JH. Interaction of CSR1 with XIAP reverses inhibition of caspases and accelerates cell death. Am J Pathol. 2012;181:463–71. 10.1016/j.ajpath.2012.04.016 22683311 PMC3409444

[37] Xiao Q Hu Y Liu Y Wang Z Geng H Hu L BEX1 promotes imatinib-induced apoptosis by binding to and antagonizing BCL-2. PLoS One. 2014;9:e91782. 10.1371/journal.pone.0091782 24626299 PMC3953594

[38] Marzo I Brenner C Zamzami N Jürgensmeier JM Susin SA Vieira HL Bax and adenine nucleotide translocator cooperate in the mitochondrial control of apoptosis. Science. 1998;281:2027–31. 10.1126/science.281.5385.2027 9748162

[39] Greenhalf W Stephan C Chaudhuri B. Role of mitochondria and C-terminal membrane anchor of Bcl-2 in Bax induced growth arrest and mortality in *Saccharomyces cerevisiae*. FEBS Lett. 1996;380:169–75. 10.1016/0014-5793(96)00044-0 8603730

[40] Carmona-Gutierrez D Bauer MA Zimmermann A Aguilera A Austriaco N Ayscough K Guidelines and recommendations on yeast cell death nomenclature. Microb Cell. 2018;5:4–31. 10.15698/mic2018.01.607 29354647 PMC5772036

[41] Krippner A Matsuno-Yagi A Gottlieb RA Babior BM. Loss of function of cytochrome c in Jurkat cells undergoing fas-mediated apoptosis. J Biol Chem. 1996;271:21629–36. 10.1074/jbc.271.35.21629 8702951

[42] Kluck RM Bossy-Wetzel E Green DR Newmeyer DD. The release of cytochrome c from mitochondria: a primary site for Bcl-2 regulation of apoptosis. Science. 1997;275:1132–6. 10.1126/science.275.5303.1132 9027315

[43] Kim CN Wang X Huang Y Ibrado AM Liu L Fang G Overexpression of Bcl-X(L) inhibits ara-C-induced mitochondrial loss of cytochrome c and other perturbations that activate the molecular cascade of apoptosis. Cancer Res. 1997;57:3115–20. 9242435

[44] Manon S Chaudhuri B Guérin M. Release of cytochrome c and decrease of cytochrome c oxidase in Bax-expressing yeast cells, and prevention of these effects by coexpression of Bcl-xL. FEBS Lett. 1997;415:29–32. 10.1016/S0014-5793(97)01087-9 9326363

[45] Rossé T Olivier R Monney L Rager M Conus S Fellay I Bcl-2 prolongs cell survival after Bax-induced release of cytochrome c. Nature. 1998;391:496–9. 10.1038/35160 9461218

[46] Priault M Camougrand N Chaudhuri B Manon S. Role of the C-terminal domain of Bax and Bcl-XL in their localization and function in yeast cells. FEBS Lett. 1999;443:225–8. 10.1016/S0014-5793(98)01661-5 9989610

[47] Priault M Cartron PF Camougrand N Antonsson B Vallette FM Manon S. Investigation of the role of the C-terminus of Bax and of tc-Bid on Bax interaction with yeast mitochondria. Cell Death Differ. 2003;10:1068–77. 10.1038/sj.cdd.4401270 12934081

[48] Polcic P Forte M. Response of yeast to the regulated expression of proteins in the Bcl-2 family. Biochem J. 2003;374:393–402. 10.1042/bj20030690 12780347 PMC1223605

[49] Schmitt E Paquet C Beauchemin M Bertrand R. Bcl-xES, a BH4- and BH2-containing antiapoptotic protein, delays Bax oligomer formation and binds Apaf-1, blocking procaspase-9 activation. Oncogene. 2004;23:3915–31. 10.1038/sj.onc.1207554 15048082

[50] Xu Q Reed JC. Bax inhibitor-1, a mammalian apoptosis suppressor identified by functional screening in yeast. Mol Cell. 1998;1:337–46. 10.1016/S1097-2765(00)80034-9 9660918

[51] Zhang H Xu Q Krajewski S Krajewska M Xie Z Fuess S BAR: an apoptosis regulator at the intersection of caspases and Bcl-2 family proteins. Proc Natl Acad Sci U S A. 2000;97:2597–602. 10.1073/pnas.97.6.2597 10716992 PMC15974

[52] Brezniceanu ML Völp K Bösser S Solbach C Lichter P Joos S HMGB1 inhibits cell death in yeast and mammalian cells and is abundantly expressed in human breast carcinoma. FASEB J. 2003;17:1295–7. 10.1096/fj.02-0621fje 12759333

[53] Takahashi Y Karbowski M Yamaguchi H Kazi A Wu J Sebti SM Loss of Bif-1 suppresses Bax/Bak conformational change and mitochondrial apoptosis. Mol Cell Biol. 2005;25:9369–82. 10.1128/MCB.25.21.9369-9382.2005 16227588 PMC1265816

[54] Khoury CM Yang Z Ismail S Greenwood MT. Characterization of a novel alternatively spliced human transcript encoding an N-terminally truncated Vps24 protein that suppresses the effects of Bax in an ESCRT independent manner in yeast. Gene. 2007;391:233–41. 10.1016/j.gene.2006.12.039 17331679

[55] Khoury CM Yang Z Li XY Vignali M Fields S Greenwood MT. A TSC22-like motif defines a novel antiapoptotic protein family. FEMS Yeast Res. 2008;8:540–63. 10.1111/j.1567-1364.2008.00367.x 18355271 PMC2593406

[56] Woo IS Jang HS Eun SY Kim HJ Ham SA Kim HJ Ran suppresses paclitaxel-induced apoptosis in human glioblastoma cells. Apoptosis. 2008;13:1223–31. 10.1007/s10495-008-0247-0 18690538

[57] Woo IS Jin H Kang ES Kim HJ Lee JH Chang KC TMEM14A inhibits *N*-(4-hydroxyphenyl) retinamide-induced apoptosis through the stabilization of mitochondrial membrane potential. Cancer Lett. 2011;309:190–8. 10.1016/j.canlet.2011.05.031 21723035

[58] Clapp C Portt L Khoury C Sheibani S Norman G Ebner P 14–3–3 protects against stress-induced apoptosis. Cell Death Dis. 2012;3:e348. 10.1038/cddis.2012.90 22785534 PMC3406589

[59] Chae HJ Ke N Kim HR Chen S Godzik A Dickman M Evolutionarily conserved cytoprotection provided by Bax inhibitor-1 homologs from animals, plants, and yeast. Gene. 2003;323:101–13. 10.1016/j.gene.2003.09.011 14659883

[60] Kampranis SC Damianova R Atallah M Toby G Kondi G Tsichlis PN A novel plant glutathione *S*-transferase/peroxidase suppresses Bax lethality in yeast. J Biol Chem. 2000;275:29207–16. 10.1074/jbc.M002359200 10859306

[61] Sanchez P de Torres Zabala M Grant M. AtBI-1, a plant homologue of Bax inhibitor-1, suppresses Bax-induced cell death in yeast and is rapidly upregulated during wounding and pathogen challenge. Plant J. 2000;21:393–9. 10.1046/j.1365-313x.2000.00690.x 10758491

[62] Pan L Kawai M Yu LH Kim KM Hirata A Umeda M The *Arabidopsis thaliana* ethylene-responsive element binding protein (AtEBP) can function as a dominant suppressor of Bax-induced cell death of yeast. FEBS Lett. 2001;508:375–8. 10.1016/S0014-5793(01)03098-8 11728455

[63] Levine A Belenghi B Damari-Weisler H Granot D. Vesicle-associated membrane protein of *Arabidopsis* suppresses Bax-induced apoptosis in yeast downstream of oxidative burst. J Biol Chem. 2001;276:46284–9. 10.1074/jbc.M107375200 11551960

[64] Chen S Vaghchhipawala Z Li W Asard H Dickman MB. Tomato phospholipid hydroperoxide glutathione peroxidase inhibits cell death induced by Bax and oxidative stresses in yeast and plants. Plant Physiol. 2004;135:1630–41. 10.1104/pp.103.038091 15235116 PMC519077

[65] Ogawa T Pan L Kawai-Yamada M Yu LH Yamamura S Koyama T Functional analysis of *Arabidopsis* ethylene-responsive element binding protein conferring resistance to Bax and abiotic stress-induced plant cell death. Plant Physiol. 2005;138:1436–45. 10.1104/pp.105.063586 15980186 PMC1176415

[66] Kim KM Jun DY Kim SK Kim CK Kim BO Kim YH Identification of novel mitochondrial membrane protein (Cdf 3) from *Arabidopsis thaliana* and its functional analysis in a yeast system. J Microbiol Biotechnol. 2007;17:891–6. 18050905

[67] Laloux G Deghelt M de Barsy M Letesson JJ De Bolle X. Identification of the essential *Brucella melitensis* porin Omp2b as a suppressor of Bax-induced cell death in yeast in a genome-wide screening. PLoS One. 2010;5:e13274. 10.1371/journal.pone.0013274 20949000 PMC2952587

[68] Hückelhoven R. BAX inhibitor-1, an ancient cell death suppressor in animals and plants with prokaryotic relatives. Apoptosis. 2004;9:299–307. 10.1023/B:APPT.0000025806.71000.1c 15258461

[69] Odat O Matta S Khalil H Kampranis SC Pfau R Tsichlis PN Old yellow enzymes, highly homologous FMN oxidoreductases with modulating roles in oxidative stress and programmed cell death in yeast. J Biol Chem. 2007;282:36010–23. 10.1074/jbc.M704058200 17897954

[70] Horowitz A Lapointe JF Eid R Sheibani S Gharib N Jones NK The human septin7 and the yeast CDC10 septin prevent Bax and copper mediated cell death in yeast. Biochim Biophys Acta. 2013;1833:3186–94. 10.1016/j.bbamcr.2013.09.004 24055994

[71] Priault M Bessoule JJ Grelaud-Coq A Camougrand N Manon S. Bax-induced cell death in yeast depends on mitochondrial lipid oxidation. Eur J Biochem. 2002;269:5440–50. 10.1046/j.1432-1033.2002.03234.x 12423342

[72] Manon S. Utilization of yeast to investigate the role of lipid oxidation in cell death. Antioxid Redox Signal. 2004;6:259–67. 10.1089/152308604322899323 15025927

[73] Kiššová I Plamondon LT Brisson L Priault M Renouf V Schaeffer J Evaluation of the roles of apoptosis, autophagy, and mitophagy in the loss of plating efficiency induced by Bax expression in yeast. J Biol Chem. 2006;281:36187–97. 10.1074/jbc.M607444200 16990272

[74] Madeo F Fröhlich E Fröhlich KU. A yeast mutant showing diagnostic markers of early and late apoptosis. J Cell Biol. 1997;139:729–34. 10.1083/jcb.139.3.729 9348289 PMC2141703

[75] Ligr M Madeo F Fröhlich E Hilt W Fröhlich KU Wolf DH. Mammalian Bax triggers apoptotic changes in yeast. FEBS Lett. 1998;438:61–5. 10.1016/S0014-5793(98)01227-7 9821959

[76] Madeo F Fröhlich E Ligr M Grey M Sigrist SJ Wolf DH Oxygen stress: a regulator of apoptosis in yeast. J Cell Biol. 1999;145:757–67. 10.1083/jcb.145.4.757 10330404 PMC2133192

[77] Ludovico P Rodrigues F Almeida A Silva MT Barrientos A Côrte-Real M. Cytochrome c release and mitochondria involvement in programmed cell death induced by acetic acid in *Saccharomyces cerevisiae*. Mol Biol Cell. 2002;13:2598–606. 10.1091/mbc.e01-12-0161 12181332 PMC117928

[78] Huh GH Damsz B Matsumoto TK Reddy MP Rus AM Ibeas JI Salt causes ion disequilibrium- induced programmed cell death in yeast and plants. Plant J. 2002;29:649–59. 10.1046/j.0960-7412.2001.01247.x 11874577

[79] Severin FF Hyman AA. Pheromone induces programmed cell death in *S. cerevisiae*. Curr Biol. 2002;12:R233–5. 10.1016/S0960-9822(02)00776-5 11937036

[80] Pozniakovsky AI Knorre DA Markova OV Hyman AA Skulachev VP Severin FF. Role of mitochondria in the pheromone- and amiodarone-induced programmed death of yeast. J Cell Biol. 2005;168:257–69. 10.1083/jcb.200408145 15657396 PMC2171581

[81] Laun P Pichova A Madeo F Fuchs J Ellinger A Kohlwein S Aged mother cells of *Saccharomyces cerevisiae* show markers of oxidative stress and apoptosis. Mol Microbiol. 2001;39:1166–73. 10.1111/j.1365-2958.2001.02317.x 11251834

[82] Hauptmann P Riel C Kunz-Schughart LA Fröhlich KU Madeo F Lehle L. Defects in *N*-glycosylation induce apoptosis in yeast. Mol Microbiol. 2006;59:765–78. 10.1111/j.1365-2958.2005.04981.x 16420350

[83] Grosfeld EV Bidiuk VA Mitkevich OV Ghazy ESMO Kushnirov VV Alexandrov AI. A systematic survey of characteristic features of yeast cell death triggered by external factors. J Fungi (Basel). 2021;7:886. 10.3390/jof7110886 34829175 PMC8626022

[84] Wadskog I Maldener C Proksch A Madeo F Adler L. Yeast lacking the SRO7/SOP1-encoded tumor suppressor homologue show increased susceptibility to apoptosis-like cell death on exposure to NaCl stress. Mol Biol Cell. 2004;15:1436–44. 10.1091/mbc.e03-02-0114 14718573 PMC363166

[85] Mazzoni C Palermo V Torella M Falcone C. HIR1, the co-repressor of histone gene transcription of *Saccharomyces cerevisiae*, acts as a multicopy suppressor of the apoptotic phenotypes of the *LSM4* mRNA degradation mutant. FEMS Yeast Res. 2005;5:1229–35. 10.1016/j.femsyr.2005.07.007 16169287

[86] Lamour V Lécluse Y Desmaze C Spector M Bodescot M Aurias A A human homolog of the S. *cerevisiae* HIR1 and HIR2 transcriptional repressors cloned from the DiGeorge syndrome critical region. Hum Mol Genet. 1995;4:791–9. 10.1093/hmg/4.5.791 7633437

[87] Guérin R Arseneault G Dumont S Rokeach LA. Calnexin is involved in apoptosis induced by endoplasmic reticulum stress in the fission yeast. Mol Biol Cell. 2008;19:4404–20. 10.1091/mbc.e08-02-0188 18701708 PMC2555920

[88] Mazzoni C Torella M Petrera A Palermo V Falcone C. PGK1, the gene encoding the glycolitic enzyme phosphoglycerate kinase, acts as a multicopy suppressor of apoptotic phenotypes in *S. cerevisiae*. Yeast. 2009;26:31–7. 10.1002/yea.1647 19180641

[89] Büttner S Ruli D Vögtle FN Galluzzi L Moitzi B Eisenberg T A yeast BH3-only protein mediates the mitochondrial pathway of apoptosis. EMBO J. 2011;30:2779–92. 10.1038/emboj.2011.197 21673659 PMC3160254

[90] Cebulski J Malouin J Pinches N Cascio V Austriaco N. Yeast Bax inhibitor, Bxi1p, is an ER-localized protein that links the unfolded protein response and programmed cell death in *Saccharomyces cerevisiae*. PLoS One. 2011;6:e20882. 10.1371/journal.pone.0020882 21673967 PMC3108976

[91] Henke N Lisak DA Schneider L Habicht J Pergande M Methner A. The ancient cell death suppressor BAX inhibitor-1. Cell Calcium. 2011;50:251-60. 10.1016/j.ceca.2011.05.005 21663964

[92] Robinson KS Clements A Williams AC Berger CN Frankel G. Bax inhibitor 1 in apoptosis and disease. Oncogene. 2011;30:2391–400. 10.1038/onc.2010.636 21297665

[93] Boise LH González-García M Postema CE Ding L Lindsten T Turka LA *Bcl-x*, a bcl-2-related gene that functions as a dominant regulator of apoptotic cell death. Cell. 1993;74:597–608. 10.1016/0092-8674(93)90508-N 8358789

[94] Kirsch DG Doseff A Chau BN Lim DS de Souza-Pinto NC Hansford R Caspase-3-dependent cleavage of Bcl-2 promotes release of cytochrome c. J Biol Chem. 1999;274:21155–61. 10.1074/jbc.274.30.21155 10409669

[95] Kale J Kutuk O Brito GC Andrews TS Leber B Letai A Phosphorylation switches Bax from promoting to inhibiting apoptosis thereby increasing drug resistance. EMBO Rep. 2018;19:e45235. 10.15252/embr.201745235 29987135 PMC6123645

[96] Alvarez-Paggi D Hannibal L Castro MA Oviedo-Rouco S Demicheli V Tórtora V Multifunctional cytochrome c: learning new tricks from an old dog. Chem Rev. 2017;117:13382–460. 10.1021/acs.chemrev.7b00257 29027792

[97] Ow YP Green DR Hao Z Mak TW. Cytochrome c: functions beyond respiration. Nat Rev Mol Cell Biol. 2008;9:532–42. 10.1038/nrm2434 18568041

[98] Brown GC Borutaite V. Regulation of apoptosis by the redox state of cytochrome c. Biochim Biophys Acta. 2008;1777:877–81. 10.1016/j.bbabio.2008.03.024 18439415

[99] Mazzoni C Falcone C. Caspase-dependent apoptosis in yeast. Biochim Biophys Acta. 2008;1783:1320–7. 10.1016/j.bbamcr.2008.02.015 18355456

[100] Váchová L Palková Z. Caspases in yeast apoptosis-like death: facts and artefacts. FEMS Yeast Res. 2007;7:12–21. 10.1111/j.1567-1364.2006.00137.x 17311581

[101] Wissing S Ludovico P Herker E Büttner S Engelhardt SM Decker T An AIF orthologue regulates apoptosis in yeast. J Cell Biol. 2004;166:969–74. 10.1083/jcb.200404138 15381687 PMC2172025

[102] Büttner S Eisenberg T Carmona-Gutierrez D Ruli D Knauer H Ruckenstuhl C Endonuclease G regulates budding yeast life and death. Mol Cell. 2007;25:233–46. 10.1016/j.molcel.2006.12.021 17244531

[103] Walter D Wissing S Madeo F Fahrenkrog B. The inhibitor-of-apoptosis protein Bir1p protects against apoptosis in *S. cerevisiae* and is a substrate for the yeast homologue of Omi/HtrA2. J Cell Sci. 2006;119:1843–51. 10.1242/jcs.02902 16608876

[104] Azzopardi M Farrugia G Balzan R. Cell-cycle involvement in autophagy and apoptosis in yeast. Mech Ageing Dev. 2017;161:211–24. 10.1016/j.mad.2016.07.006 27450768

[105] Kang R Zeh HJ Lotze MT Tang D. The Beclin 1 network regulates autophagy and apoptosis. Cell Death Differ. 2011;18:571–80. 10.1038/cdd.2010.191 21311563 PMC3131912

[106] Falcone C Mazzoni C. RNA stability and metabolism in regulated cell death, aging and diseases. FEMS Yeast Res. 2018;18. 10.1093/femsyr/foy050 29986027

[107] Ring J Sommer C Carmona-Gutierrez D Ruckenstuhl C Eisenberg T Madeo F. The metabolism beyond programmed cell death in yeast. Exp Cell Res. 2012;318:1193–200. 10.1016/j.yexcr.2012.03.019 22480867 PMC3396845

[108] Guaragnella N Palermo V Galli A Moro L Mazzoni C Giannattasio S. The expanding role of yeast in cancer research and diagnosis: insights into the function of the oncosuppressors p53 and BRCA1/2. FEMS Yeast Res. 2014;14:2–16. 10.1111/1567-1364.12094 24103154

[109] Kaczanowski S Klim J Zielenkiewicz U. An apoptotic and endosymbiotic explanation of the Warburg and the Inverse Warburg Hypotheses. Int J Mol Sci. 2018;19:3100. 10.3390/ijms19103100 30308966 PMC6213112

[110] Furuya N Yu J Byfield M Pattingre S Levine B. The evolutionarily conserved domain of Beclin 1 is required for Vps34 binding, autophagy and tumor suppressor function. Autophagy. 2005;1:46–52. 10.4161/auto.1.1.1542 16874027

[111] Murakawa T Yamaguchi O Hashimoto A Hikoso S Takeda T Oka T Bcl-2-like protein 13 is a mammalian Atg32 homologue that mediates mitophagy and mitochondrial fragmentation. Nat Commun. 2015;6:7527. 10.1038/ncomms8527 26146385 PMC4501433

[112] Kataoka T Holler N Micheau O Martinon F Tinel A Hofmann K Bcl-rambo, a novel Bcl-2 homologue that induces apoptosis via its unique C-terminal extension. J Biol Chem. 2001;276:19548–54. 10.1074/jbc.M010520200 11262395

[113] Pattingre S Levine B. Bcl-2 inhibition of autophagy: a new route to cancer? Cancer Res. 2006;66:2885–8. 10.1158/0008-5472.CAN-05-4412 16540632

[114] Gourlay CW Du W Ayscough KR. Apoptosis in yeast–mechanisms and benefits to a unicellular organism. Mol Microbiol. 2006;62:1515–21. 10.1111/j.1365-2958.2006.05486.x 17087770

[115] Váchová L Palková Z. Physiological regulation of yeast cell death in multicellular colonies is triggered by ammonia. J Cell Biol. 2005;169:711–7. 10.1083/jcb.200410064 15939758 PMC2171614

[116] Palková Z Váchová L. Life within a community: benefit to yeast long-term survival. FEMS Microbiol Rev. 2006;30:806–24. 10.1111/j.1574-6976.2006.00034.x 16911045

[117] Zha H Fisk HA Yaffe MP Mahajan N Herman B Reed JC. Structure-function comparisons of the proapoptotic protein Bax in yeast and mammalian cells. Mol Cell Biol. 1996;16:6494–508. 10.1128/MCB.16.11.6494 8887678 PMC231651

[118] Pavlov EV Priault M Pietkiewicz D Cheng EH Antonsson B Manon S A novel, high conductance channel of mitochondria linked to apoptosis in mammalian cells and Bax expression in yeast. J Cell Biol. 2001;155:725–31. 10.1083/jcb.200107057 11724814 PMC2150879

[119] Tremblais K Oliver L Juin P Le Cabellec TM Meflah K Vallette FM. The C-terminus of bax is not a membrane addressing/anchoring signal. Biochem Biophys Res Commun. 1999;260:582–91. 10.1006/bbrc.1999.0904 10403809

[120] Oliver L Priault M Tremblais K LeCabellec M Meflah K Manon S The substitution of the C-terminus of bax by that of bcl-xL does not affect its subcellular localization but abrogates its pro-apoptotic properties. FEBS Lett. 2000;487:161–5. 10.1016/S0014-5793(00)02330-9 11150501

[121] Arokium H Camougrand N Vallette FM Manon S. Studies of the interaction of substituted mutants of BAX with yeast mitochondria reveal that the C-terminal hydrophobic alpha-helix is a second ART sequence and plays a role in the interaction with anti-apoptotic BCL-xL. J Biol Chem. 2004;279:52566–73. 10.1074/jbc.M408373200 15459197

[122] Cartron PF Arokium H Oliver L Meflah K Manon S Vallette FM. Distinct domains control the addressing and the insertion of Bax into mitochondria. J Biol Chem. 2005;280:10587–98. 10.1074/jbc.M409714200 15590655

[123] Simonyan L Légiot A Lascu I Durand G Giraud MF Gonzalez C The substitution of proline 168 favors Bax oligomerization and stimulates its interaction with LUVs and mitochondria. Biochim Biophys Acta Biomembr. 2017;1859:1144–55. 10.1016/j.bbamem.2017.03.010 28322731

[124] Suzuki M Youle RJ Tjandra N. Structure of Bax: coregulation of dimer formation and intracellular localization. Cell. 2000;103:645–54. 10.1016/S0092-8674(00)00167-7 11106734

[125] Gardai SJ Hildeman DA Frankel SK Whitlock BB Frasch SC Borregaard N Phosphorylation of Bax Ser184 by Akt regulates its activity and apoptosis in neutrophils. J Biol Chem. 2004;279:21085–95. 10.1074/jbc.M400063200 14766748

[126] Arokium H Ouerfelli H Velours G Camougrand N Vallette FM Manon S. Substitutions of potentially phosphorylatable serine residues of Bax reveal how they may regulate its interaction with mitochondria. J Biol Chem. 2007;282:35104–12. 10.1074/jbc.M704891200 17911107

[127] Schellenberg B Wang P Keeble JA Rodriguez-Enriquez R Walker S Owens TW Bax exists in a dynamic equilibrium between the cytosol and mitochondria to control apoptotic priming. Mol Cell. 2013;49:959–71. 10.1016/j.molcel.2012.12.022 23375500 PMC3594749

[128] Simonyan L Renault TT Novais MJ Sousa MJ Côrte-Real M Camougrand N Regulation of Bax/mitochondria interaction by AKT. FEBS Lett. 2016;590:13–21. 10.1002/1873-3468.12030 26763134

[129] Wang Q Sun SY Khuri F Curran WJ Deng X. Mono- or double-site phosphorylation distinctly regulates the proapoptotic function of Bax. PLoS One. 2010;5:e13393. 10.1371/journal.pone.0013393 20976235 PMC2954808

[130] Garenne D Renault TT Manon S. Bax mitochondrial relocation is linked to its phosphorylation and its interaction with Bcl-xL. Microb Cell. 2016;3:597–605. 10.15698/mic2016.12.547 28357332 PMC5348979

[131] Cartron PF Oliver L Martin S Moreau C LeCabellec MT Jezequel P The expression of a new variant of the pro-apoptotic molecule Bax, Baxpsi, is correlated with an increased survival of glioblastoma multiforme patients. Hum Mol Genet. 2002;11:675–87. 10.1093/hmg/11.6.675 11912183

[132] Goping IS Gross A Lavoie JN Nguyen M Jemmerson R Roth K Regulated targeting of BAX to mitochondria. J Cell Biol. 1998;143:207–15. 10.1083/jcb.143.1.207 9763432 PMC2132805

[133] Cartron PF Moreau C Oliver L Mayat E Meflah K Vallette FM. Involvement of the N-terminus of Bax in its intracellular localization and function. FEBS Lett. 2002;512:95–100. 10.1016/S0014-5793(02)02227-5 11852059

[134] Cartron PF Priault M Oliver L Meflah K Manon S Vallette FM. The N-terminal end of Bax contains a mitochondrial-targeting signal. J Biol Chem. 2003;278:11633–41. 10.1074/jbc.M208955200 12529375

[135] Bellot G Cartron PF Er E Oliver L Juin P Armstrong LC TOM22, a core component of the mitochondria outer membrane protein translocation pore, is a mitochondrial receptor for the proapoptotic protein Bax. Cell Death Differ. 2007;14:785–94. 10.1038/sj.cdd.4402055 17096026

[136] Ott M Norberg E Walter KM Schreiner P Kemper C Rapaport D The mitochondrial TOM complex is required for tBid/Bax-induced cytochrome c release. J Biol Chem. 2007;282:27633–9. 10.1074/jbc.M703155200 17635912

[137] Cartron PF Bellot G Oliver L Grandier-Vazeille X Manon S Vallette FM. Bax inserts into the mitochondrial outer membrane by different mechanisms. FEBS Lett. 2008;582:3045–51. 10.1016/j.febslet.2008.07.047 18687331

[138] Sanjuán Szklarz LK Kozjak-Pavlovic V Vögtle FN Chacinska A Milenkovic D Vogel S Preprotein transport machineries of yeast mitochondrial outer membrane are not required for Bax-induced release of intermembrane space proteins. J Mol Biol. 2007;368:44–54. 10.1016/j.jmb.2007.01.016 17335847

[139] Renault TT Grandier-Vazeille X Arokium H Velours G Camougrand N Priault M The cytosolic domain of human Tom22 modulates human Bax mitochondrial translocation and conformation in yeast. FEBS Lett. 2012;586:116–21. 10.1016/j.febslet.2011.12.003 22198199

[140] Sandow JJ Tan IK Huang AS Masaldan S Bernardini JP Wardak AZ Dynamic reconfiguration of pro-apoptotic BAK on membranes. EMBO J. 2021;40:e107237. 10.15252/embj.2020107237 34523147 PMC8521275

[141] Motz C Martin H Krimmer T Rassow J. Bcl-2 and porin follow different pathways of TOM-dependent insertion into the mitochondrial outer membrane. J Mol Biol. 2002;323:729–38. 10.1016/S0022-2836(02)00995-6 12419260

[142] Kaufmann T Schlipf S Sanz J Neubert K Stein R Borner C. Characterization of the signal that directs Bcl-x(L), but not Bcl-2, to the mitochondrial outer membrane. J Cell Biol. 2003;160:53–64. 10.1083/jcb.200210084 12515824 PMC2172731

[143] Lalier L Mignard V Joalland MP Lanoé D Cartron PF Manon S TOM20-mediated transfer of Bcl2 from ER to MAM and mitochondria upon induction of apoptosis. Cell Death Dis. 2021;12:182. 10.1038/s41419-021-03471-8 33589622 PMC7884705

[144] Frank DO Dengjel J Wilfling F Kozjak-Pavlovic V Häcker G Weber A. The pro-apoptotic BH3-only protein Bim interacts with components of the translocase of the outer mitochondrial membrane (TOM). PLoS One. 2015;10:e0123341. 10.1371/journal.pone.0123341 25875815 PMC4398440

[145] Vance JE. MAM (mitochondria-associated membranes) in mammalian cells: lipids and beyond. Biochim Biophys Acta. 2014;1841:595–609. 10.1016/j.bbalip.2013.11.014 24316057

[146] Vance JE. Phospholipid synthesis and transport in mammalian cells. Traffic. 2015;16:1–18. 10.1111/tra.12230 25243850

[147] Lang A John Peter AT Kornmann B. ER-mitochondria contact sites in yeast: beyond the myths of ERMES. Curr Opin Cell Biol. 2015;35:7–12. 10.1016/j.ceb.2015.03.002 25836730

[148] Légiot A Céré C Dupoiron T Kaabouni M Camougrand N Manon S. Mitochondria-associated membranes (MAMs) are involved in Bax mitochondrial localization and cytochrome c release. Microb Cell. 2019;6:257–66. 10.15698/mic2019.05.678 31114795 PMC6506693

[149] Edlich F Banerjee S Suzuki M Cleland MM Arnoult D Wang C Bcl-x(L) retrotranslocates Bax from the mitochondria into the cytosol. Cell. 2011;145:104–16. 10.1016/j.cell.2011.02.034 21458670 PMC3070914

[150] Todt F Cakir Z Reichenbach F Youle RJ Edlich F. The C-terminal helix of Bcl-x(L) mediates Bax retrotranslocation from the mitochondria. Cell Death Differ. 2013;20:333–42. 10.1038/cdd.2012.131 23079612 PMC3554327

[151] Renault TT Teijido O Missire F Ganesan YT Velours G Arokium H Bcl-xL stimulates Bax relocation to mitochondria and primes cells to ABT-737. Int J Biochem Cell Biol. 2015;64:136–46. 10.1016/j.biocel.2015.03.020 25862283

[152] Renault TT Dejean LM Manon S. A brewing understanding of the regulation of Bax function by Bcl-xL and Bcl-2. Mech Ageing Dev. 2017;161:201–10. 10.1016/j.mad.2016.04.007 27112371

[153] Shimizu S Narita M Tsujimoto Y. Bcl-2 family proteins regulate the release of apoptogenic cytochrome c by the mitochondrial channel VDAC. Nature. 1999;399:483–7. 10.1038/20959 10365962

[154] Priault M Chaudhuri B Clow A Camougrand N Manon S. Investigation of bax-induced release of cytochrome c from yeast mitochondria permeability of mitochondrial membranes, role of VDAC and ATP requirement. Eur J Biochem. 1999;260:684–91. 10.1046/j.1432-1327.1999.00198.x 10102996

[155] Gross A Pilcher K Blachly-Dyson E Basso E Jockel J Bassik MC Biochemical and genetic analysis of the mitochondrial response of yeast to BAX and BCL-X(L). Mol Cell Biol. 2000;20:3125–36. 10.1128/MCB.20.9.3125-3136.2000 10757797 PMC85607

[156] Baines CP Kaiser RA Sheiko T Craigen WJ Molkentin JD. Voltage-dependent anion channels are dispensable for mitochondrial-dependent cell death. Nat Cell Biol. 2007;9:550–5. 10.1038/ncb1575 17417626 PMC2680246

[157] Boorstein WR Ziegelhoffer T Craig EA. Molecular evolution of the HSP70 multigene family. J Mol Evol. 1994;38:1–17. 10.1007/BF00175490 8151709

[158] Murphy ME. The HSP70 family and cancer. Carcinogenesis. 2013;34:1181–8. 10.1093/carcin/bgt111 23563090 PMC3670260

[159] Guo Z Song T Wang Z Lin D Cao K Liu P The chaperone Hsp70 is a BH3 receptor activated by the pro-apoptotic Bim to stabilize anti-apoptotic clients. J Biol Chem. 2020;295:12900–9. 10.1074/jbc.RA120.013364 32651234 PMC7489912

[160] Pan H Song T Wang Z Guo Y Zhang H Ji T Ectopic BH3-only protein Bim acts as a cochaperone to positively regulate Hsp70 in yeast. J Biochem. 2021;170:539–45. 10.1093/jb/mvab073 34185080

[161] Matsuyama S Xu Q Velours J Reed JC. The mitochondrial F0F1-ATPase proton pump is required for function of the proapoptotic protein Bax in yeast and mammalian cells. Mol Cell. 1998;1:327–36. 10.1016/S1097-2765(00)80033-7 9660917

[162] Prudent J Popgeorgiev N Bonneau B Thibaut J Gadet R Lopez J Bcl-wav and the mitochondrial calcium uniporter drive gastrula morphogenesis in zebrafish. Nat Commun. 2013;4:2330. 10.1038/ncomms3330 23942336

[163] Popgeorgiev N Sa JD Jabbour L Banjara S Nguyen TTM Akhavan-E-Sabet A Ancient and conserved functional interplay between Bcl-2 family proteins in the mitochondrial pathway of apoptosis. Sci Adv. 2020;6:eabc4149. 10.1126/sciadv.abc4149 32998881 PMC7527217

[164] Almeida B Silva A Mesquita A Sampaio-Marques B Rodrigues F Ludovico P. Drug-induced apoptosis in yeast. Biochim Biophys Acta. 2008;1783:1436–48. 10.1016/j.bbamcr.2008.01.005 18252203

[165] Verbandt S Cammue BPA Thevissen K. Yeast as a model for the identification of novel survival- promoting compounds applicable to treat degenerative diseases. Mech Ageing Dev. 2017;161:306–16. 10.1016/j.mad.2016.06.003 27287065

[166] Pereira C Lopes-Rodrigues V Coutinho I Neves MP Lima RT Pinto M Potential small-molecule activators of caspase-7 identified using yeast-based caspase-3 and -7 screening assays. Eur J Pharm Sci. 2014;54:8–16. 10.1016/j.ejps.2013.12.017 24398107

[167] Soares J Pereira NA Monteiro Â Leão M Bessa C Dos Santos DJ Oxazoloisoindolinones with *in vitro* antitumor activity selectively activate a p53-pathway through potential inhibition of the p53-MDM2 interaction. Eur J Pharm Sci. 2015;66:138–47. 10.1016/j.ejps.2014.10.006 25312347

[168] Soares J Raimundo L Pereira NA Monteiro Â Gomes S Bessa C Reactivation of wild-type and mutant p53 by tryptophanolderived oxazoloisoindolinone SLMP53-1, a novel anticancer small- molecule. Oncotarget. 2016;7:4326–43. 10.18632/oncotarget.6775 26735173 PMC4826208

[169] Gautier F Guillemin Y Cartron PF Gallenne T Cauquil N Le Diguarher T Bax activation by engagement with, then release from, the BH3 binding site of Bcl-xL. Mol Cell Biol. 2011;31:832–44. 10.1128/MCB.00161-10 21173168 PMC3028639

[170] Hohmann S. Nobel yeast research. FEMS Yeast Res. 2016;16:fow094. 10.1093/femsyr/fow094 27770011

[171] Gonzalvez F Bessoule JJ Rocchiccioli F Manon S Petit PX. Role of cardiolipin on tBid and tBid/Bax synergistic effects on yeast mitochondria. Cell Death Differ. 2005;12:659–67. 10.1038/sj.cdd.4401585 15818414

[172] Guscetti F Nath N Denko N. Functional characterization of human proapoptotic molecules in yeast S. *cerevisiae*. FASEB J. 2005;19:464–6. 10.1096/fj.04-2316fje 15632273

[173] Gallenne T Gautier F Oliver L Hervouet E Noël B Hickman JA Bax activation by the BH3-only protein Puma promotes cell dependence on antiapoptotic Bcl-2 family members. J Cell Biol. 2009;185:279–90. 10.1083/jcb.200809153 19380879 PMC2700382

[174] Gérecová G Kopanicová J Jaká P Běhalová L Juhásová B Bhatia-Kiššová I BH3-only proteins Noxa, Bik, Bmf, and Bid activate Bax and Bak indirectly when studied in yeast model. FEMS Yeast Res. 2013;13:747–54. 10.1111/1567-1364.12074 23991648

[175] Hanada M Aimé-Sempé C Sato T Reed JC. Structure-function analysis of Bcl-2 protein. Identification of conserved domains important for homodimerization with Bcl-2 and heterodimerization with Bax. J Biol Chem. 1995;270:11962–9. 10.1074/jbc.270.20.11962 7744846

[176] Zhang H Cowan-Jacob SW Simonen M Greenhalf W Heim J Meyhack B. Structural basis of BFL-1 for its interaction with BAX and its anti-apoptotic action in mammalian and yeast cells. J Biol Chem. 2000;275:11092–9. 10.1074/jbc.275.15.11092 10753914

[177] Juhásová B Bhatia-Kiššová I Polčicová K Mentel M Forte M Polčic P. Reconstitution of interactions of murine gammaherpesvirus 68 M11 with Bcl-2 family proteins in yeast. Biochem Biophys Res Commun. 2011;407:783–7. 10.1016/j.bbrc.2011.03.100 21439939

[178] Banadyga L Lam SC Okamoto T Kvansakul M Huang DC Barry M. Deerpox virus encodes an inhibitor of apoptosis that regulates Bak and Bax. J Virol. 2011;85:1922–34. 10.1128/JVI.01959-10 21159883 PMC3067780

[179] Kawai M Pan L Reed JC Uchimiya H. Evolutionally conserved plant homologue of the Bax inhibitor-1 (*BI-1*) gene capable of suppressing Bax-induced cell death in yeast^1^. FEBS Lett. 1999;464:143–7. 10.1016/S0014-5793(99)01695-6 10618494

[180] Bolduc N Ouellet M Pitre F Brisson LF. Molecular characterization of two plant BI-1 homologues which suppress Bax-induced apoptosis in human 293 cells. Planta. 2003;216:377–86. 10.1007/s00425-002-0879-1 12520328

[181] Akintade DD Chaudhuri B. Human VAMP3 suppresses or negatively regulates Bax induced apoptosis in yeast. Biomedicines. 2021;9:95. 10.3390/biomedicines9010095 33478086 PMC7835773

[182] Derf A Sharma A Bharate SB Chaudhuri B. Aegeline, a natural product from the plant Aegle marmelos, mimics the yeast SNARE protein Sec22p in suppressing α-synuclein and Bax toxicity in yeast. Bioorg Med Chem Lett. 2019;29:454–60. 10.1016/j.bmcl.2018.12.028 30579794

[183] Camougrand N Grelaud-Coq A Marza E Priault M Bessoule JJ Manon S. The product of the *UTH1* gene, required for Bax-induced cell death in yeast, is involved in the response to rapamycin. Mol Microbiol. 2003;47:495–506. 10.1046/j.1365-2958.2003.03311.x 12519199

[184] Manon S Priault M Camougrand N. Mitochondrial AAA-type protease Yme1p is involved in Bax effects on cytochrome c oxidase. Biochem Biophys Res Commun. 2001;289:1314–9. 10.1006/bbrc.2001.6120 11741339

[185] Alves S Neiri L Chaves SR Vieira S Trindade D Manon S N-terminal acetylation modulates Bax targeting to mitochondria. Int J Biochem Cell Biol. 2018;95:35–42. 10.1016/j.biocel.2017.12.004 29233735

[186] Sawitri WD Slameto S Sugiharto B Kim KM. Identification of Chinese cabbage sentrin as a suppressor of Bax-induced cell death in yeast. J Microbiol Biotechnol. 2012;22:600–6. 10.4014/jmb.1109.09038 22561852

[187] Li A Harris DA. Mammalian prion protein suppresses Bax-induced cell death in yeast. J Biol Chem. 2005;280:17430–4. 10.1074/jbc.C500058200 15753097

[188] Bounhar Y Mann KK Roucou X LeBlanc AC. Prion protein prevents Bax-mediated cell death in the absence of other Bcl-2 family members in *Saccharomyces cerevisiae*. FEMS Yeast Res. 2006;6:1204–12. 10.1111/j.1567-1364.2006.00122.x 17156017

[189] Akintade DD Chaudhuri B. The effect of copy number on α-synuclein’s toxicity and its protective role in Bax–induced apoptosis, in yeast. Biosci Rep. 2020;40:BSR20201912. 10.1042/BSR20201912 32794578 PMC7468099

[190] Silva RD Manon S Gonçalves J Saraiva L Côrte-Real M. Modulation of Bax mitochondrial insertion and induced cell death in yeast by mammalian protein kinase Cα. Exp Cell Res. 2011;317:781–90. 10.1016/j.yexcr.2010.12.001 21172347

[191] Rouchidane Eyitayo A Gonin M Arokium H Manon S. Contribution of yeast studies to the understanding of BCL-2 family intracellular trafficking. Int J Mol Sci. 2021;22:4086. 10.3390/ijms22084086 33920941 PMC8071328

[192] Kalderon B Kogan G Bubis E Pines O. Cytosolic Hsp60 can modulate proteasome activity in yeast. J Biol Chem. 2015;290:3542–51. 10.1074/jbc.M114.626622 25525272 PMC4319021

[193] Manzanares-Estreder S Pascual-Ahuir A Proft M. Stress-activated degradation of sphingolipids regulates mitochondrial function and cell death in yeast. Oxid Med Cell Longev. 2017;2017:2708345. 10.1155/2017/2708345 28845213 PMC5563427

[194] Iraqui I Faye G Ragu S Masurel-Heneman A Kolodner RD Huang ME. Human peroxiredoxin PrxI is an orthologue of yeast Tsa1, capable of suppressing genome instability in *Saccharomyces cerevisiae*. Cancer Res. 2008;68:1055–63. 10.1158/0008-5472.CAN-07-2683 18281480 PMC2761232

[195] Eid R Boucher E Gharib N Khoury C Arab NT Murray A Identification of human ferritin, heavy polypeptide 1 (FTH1) and yeast RGI1 (YER067W) as pro-survival sequences that counteract the effects of Bax and copper in *Saccharomyces cerevisiae*. Exp Cell Res. 2016;342:52–61. 10.1016/j.yexcr.2016.02.010 26886577

[196] Chen C Wanduragala S Becker DF Dickman MB. Tomato QM-like protein protects *Saccharomyces cerevisiae* cells against oxidative stress by regulating intracellular proline levels. Appl Environ Microbiol. 2006;72:4001–6. 10.1128/AEM.02428-05 16751508 PMC1489650

[197] Kilili KG Atanassova N Vardanyan A Clatot N Al-Sabarna K Kanellopoulos PN Differential roles of tau class glutathione *S*-transferases in oxidative stress. J Biol Chem. 2004;279:24540–51. 10.1074/jbc.M309882200 15037622

[198] Moon H Baek D Lee B Prasad DT Lee SY Cho MJ Soybean ascorbate peroxidase suppresses Bax-induced apoptosis in yeast by inhibiting oxygen radical generation. Biochem Biophys Res Commun. 2002;290:457–62. 10.1006/bbrc.2001.6208 11779192

[199] Harris MH Vander Heiden MG Kron SJ Thompson CB. Role of oxidative phosphorylation in Bax toxicity. Mol Cell Biol. 2000;20:3590–6. 10.1128/MCB.20.10.3590-3596.2000 10779348 PMC85651

[200] Eun SY Woo IS Jang HS Jin H Kim MY Kim HJ Identification of cytochrome c oxidase subunit 6A1 as a suppressor of Bax-induced cell death by yeast-based functional screening. Biochem Biophys Res Commun. 2008;373:58–63. 10.1016/j.bbrc.2008.05.178 18549809

[201] Reekmans R De Smet K Chen C Van Hummelen P Contreras R. Old yellow enzyme interferes with Bax-induced NADPH loss and lipid peroxidation in yeast. FEMS Yeast Res. 2005;5:711–25. 10.1016/j.femsyr.2004.12.010 15851100

[202] Baek D Jin Y Jeong JC Lee HJ Moon H Lee J Suppression of reactive oxygen species by glyceraldehyde-3-phosphate dehydrogenase. Phytochemistry. 2008;69:333–8. 10.1016/j.phytochem.2007.07.027 17854848

[203] Yang Z Khoury C Jean-Baptiste G Greenwood MT. Identification of mouse sphingomyelin synthase 1 as a suppressor of Bax-mediated cell death in yeast. FEMS Yeast Res. 2006;6:751–62. 10.1111/j.1567-1364.2006.00052.x 16879426

[204] Nagano M Ihara-Ohori Y Imai H Inada N Fujimoto M Tsutsumi N Functional association of cell death suppressor, Arabidopsis Bax inhibitor-1, with fatty acid 2-hydroxylation through cytochrome b_5_. Plant J. 2009;58:122–34. 10.1111/j.1365-313X.2008.03765.x 19054355

[205] Gan Y Zhang L Zhang Z Dong S Li J Wang Y The LCB2 subunit of the sphingolip biosynthesis enzyme serine palmitoyltransferase can function as an attenuator of the hypersensitive response and Bax-induced cell death. New Phytol. 2009;181:127–46. 10.1111/j.1469-8137.2008.02642.x 19076721

[206] Eid R Sheibani S Gharib N Lapointe JF Horowitz A Vali H Human ribosomal protein L9 is a Bax suppressor that promotes cell survival in yeast. FEMS Yeast Res. 2014;14:495–507. 10.1111/1567-1364.12121 24305165

